# Basilar Artery Occlusion Associated With Paradoxical Embolism After Traumatic Spinal Cord Injury: A Case Report

**DOI:** 10.7759/cureus.116615

**Published:** 2026-09-20

**Authors:** Ramón Padilla Velázquez, Irving Santiago Fraire Félix, Gustavo Saucedo Sabas, Eliseo Varela Martínez, Edmundo Israel Roque Márquez

**Affiliations:** 1 Department of Critical Care Medicine, Intensive Care Unit, Centenario Hospital Miguel Hidalgo, Aguascalientes, MEX

**Keywords:** basilar artery occlusion, mechanical thrombectomy, paradoxical embolism, patent foramen ovale, traumatic spinal cord injury

## Abstract

Basilar artery occlusion is a neurological emergency associated with high mortality and severe disability. In young patients without conventional vascular risk factors, identifying an uncommon embolic mechanism is essential for secondary stroke prevention. We report the case of a previously healthy 21-year-old man who sustained a 6-meter fall resulting in T9-T10 compression fractures with spinal canal compromise and complete traumatic spinal cord injury. He underwent posterior thoracic spinal instrumentation. During hospitalization, he developed sudden neurological deterioration characterized by a generalized tonic-clonic seizure, impaired consciousness, respiratory failure, and hemodynamic instability. Computed tomography angiography demonstrated a partial distal basilar artery occlusion without intracranial hemorrhage. Intravenous thrombolysis was contraindicated because of recent major trauma and spinal surgery. Mechanical thrombectomy using a direct aspiration technique achieved complete reperfusion (thrombolysis in cerebral infarction (TICI) scale 3). Transthoracic echocardiography with agitated saline demonstrated significant right-to-left shunting, with more than 30 microbubbles appearing in the left-sided cardiac chambers from the second cardiac cycle. Subsequent transesophageal echocardiography demonstrated a patent foramen ovale with increased interatrial septal mobility. Although deep venous thrombosis was not documented, the combination of severe spinal cord injury, prolonged immobilization, a prothrombotic state, and a patent foramen ovale with significant right-to-left shunting supported paradoxical embolism as the most plausible mechanism. This case highlights the importance of considering paradoxical embolism in young hospitalized patients who develop posterior circulation stroke following major trauma.

## Introduction

Acute basilar artery occlusion (BAO) is a severe form of ischemic stroke associated with a high risk of death and permanent neurological disability. Although it accounts for only 1-4% of all ischemic strokes, early recognition and rapid restoration of blood flow are critical to improving functional outcomes [1]. Evidence from the BEST, BASICS, ATTENTION, and BAOCHE trials, together with recommendations from the European Stroke Organisation (ESO) and the European Society for Minimally Invasive Neurological Therapy (ESMINT), supports mechanical thrombectomy as an important reperfusion strategy in carefully selected patients with posterior circulation occlusion [2-6].

In young adults, identifying the underlying etiology is particularly important when stroke occurs in the absence of conventional vascular risk factors. Paradoxical embolism occurs when thrombotic material from the venous circulation reaches the systemic arterial circulation through a right-to-left shunt. A patent foramen ovale (PFO) can provide the anatomical pathway for this mechanism, particularly in the presence of venous thromboembolic risk factors and significant right-to-left shunting [7-9].

Traumatic spinal cord injury (TSCI) creates a prothrombotic state characterized by immobilization, venous stasis, and activation of an inflammatory response, significantly increasing the risk of venous thromboembolism (VTE) during the first weeks after trauma [10-12]. However, basilar artery occlusion occurring after TSCI, with paradoxical embolism through a PFO as the probable mechanism, represents an unusual and scarcely reported clinical presentation.

We present the case of a young adult with complete TSCI who developed acute basilar artery occlusion during hospitalization and was successfully treated with mechanical thrombectomy. The etiological workup identified a PFO with significant right-to-left shunting, supporting paradoxical embolism as a plausible mechanism in the setting of substantial venous thromboembolic risk. This case highlights an uncommon potential mechanism of posterior circulation stroke after severe TSCI and emphasizes the importance of considering alternative embolic sources in young patients without conventional vascular risk factors.

## Case presentation

A 21-year-old man with no relevant medical history or family history of thrombotic events was admitted after falling approximately six meters while working, with direct impact to the dorsolumbar region. His weight was 80 kg, height 1.67 m, and body mass index was 28.7 kg/m².

On admission, his blood pressure was 146/89 mmHg and heart rate was 123 beats/minute, with sinus rhythm. He was conscious and oriented. Neurological examination showed flaccid paraplegia, loss of sensation below T10, and urinary retention, consistent with a complete TSCI (American Spinal Injury Association (ASIA) Impairment Scale [13] grade A). Spinal computed tomography showed compression fractures of T9 and T10, with retropulsion of the T10 vertebral body and narrowing of the spinal canal (Figure 1). Fractures of the posterior arches of T9 and T10, a fracture of the left 10th rib, and surrounding soft tissue edema were also observed.

**Figure 1:**
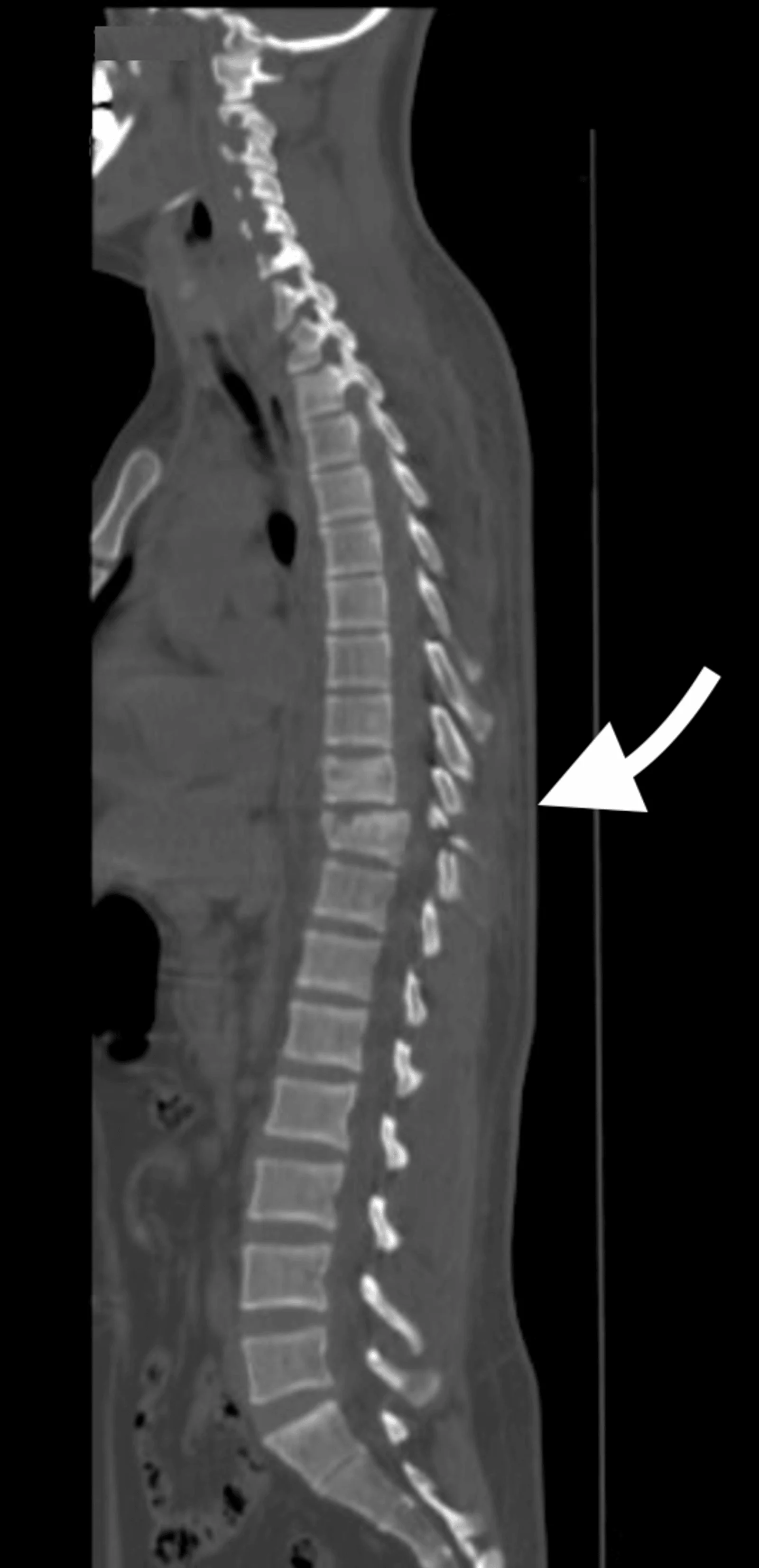
Thoracic spine CT (sagittal view) showing T9–T10 compression fractures with retropulsion of the T10 vertebral body associated to the narrowing of the spinal canal

Four days after admission, the patient underwent surgical stabilization with posterior thoracic instrumentation at T8-T9 and T11-T12. The procedure was completed without complications.

On the first postoperative day, while in the trauma ward, the patient developed sudden neurological deterioration at approximately 12:20 hours, with decreased level of consciousness, a generalized tonic-clonic seizure lasting less than one minute followed by a postictal period, respiratory distress, and hemodynamic instability. Neurological examination showed facial asymmetry and left-sided weakness, predominantly involving the face and upper limb. His pupils were equal and reactive to light, without nuchal rigidity. The National Institutes of Health Stroke Scale (NIHSS) score [14] at stroke onset was 15; however, interpretation of the score was limited by the pre-existing complete spinal cord injury (ASIA A), which accounted for bilateral lower-extremity motor deficits present before the stroke.

Electrocardiography showed sinus rhythm without significant abnormalities. Laboratory evaluation showed hemoglobin 12.3 g/dL, hematocrit 34%, leukocyte count 12.22 × 10³/µL, platelet count 168 × 10³/µL, serum creatinine 0.9 mg/dL, blood urea nitrogen 16 mg/dL, sodium 137 mmol/L, and potassium 3.6 mmol/L. Serum lactate was 1.6 mmol/L. Coagulation studies showed a prothrombin time of 14 s, activated partial thromboplastin time of 34.0 seconds, and international normalised ratio (INR) of 1.28.

An acute cerebrovascular event was suspected, and the institutional stroke protocol was activated. CT angiography (CTA) was obtained at 12:38 hours, 18 minutes after symptom onset, and showed an acute partial occlusion of the distal basilar artery without intracranial hemorrhage. The posterior circulation Alberta Stroke Program Early CT Score (pc-ASPECTS) [15] was 10. CTA showed no evidence of vertebral artery dissection or other traumatic arterial injury. Because of the recent major trauma and spinal surgery, intravenous thrombolysis was not considered a therapeutic option.

The patient was immediately transferred to the angiography suite. Arterial puncture for mechanical thrombectomy was performed at 16:07 hours. Cerebral angiography confirmed distal basilar artery occlusion, with poor filling of the distal branches and limited collateral circulation.

Mechanical thrombectomy was performed using a direct aspiration first-pass technique (ADAPT). The left vertebral artery was accessed, and an aspiration catheter was advanced to the proximal portion of the basilar artery thrombus. Complete reperfusion (thrombolysis in cerebral infarction (TICI) [16] scale 3) was achieved at 16:27 hours. Angiographic findings before and after mechanical thrombectomy are shown in Figure 2.

**Figure 2:**
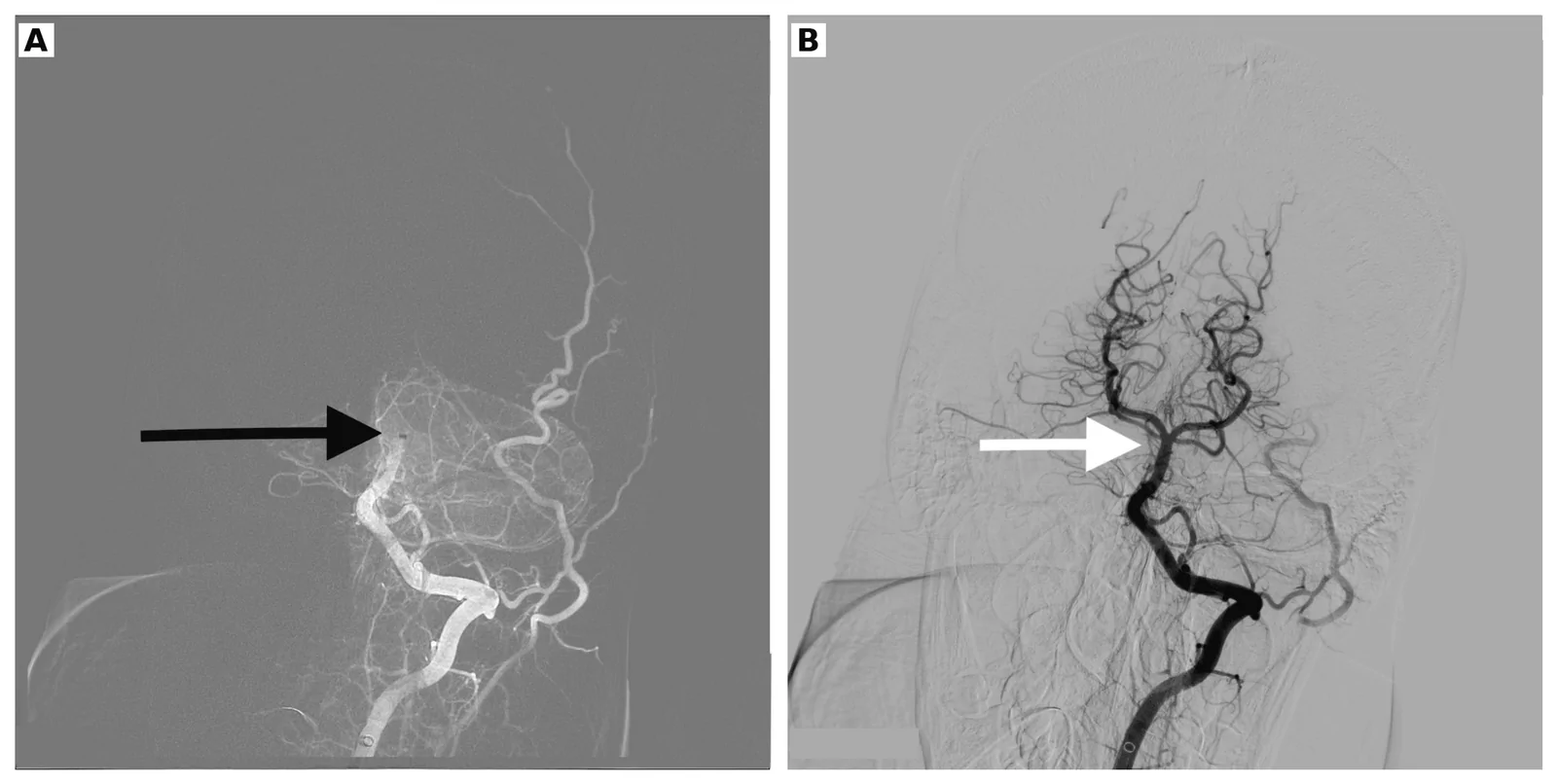
Basilar angiography before and after mechanical thrombectomy (A) Initial angiography showing distal basilar artery occlusion with impaired opacification of the distal posterior circulation (black arrow). (B) Complete reperfusion of the basilar artery after mechanical thrombectomy (white arrow), corresponding to TICI 3 reperfusion. TICI: thrombolysis in cerebral infarction scale

As part of the etiological stroke workup, continuous cardiac rhythm monitoring showed sinus rhythm without documented arrhythmias. Lower-extremity venous Doppler ultrasonography or CT venography was not performed; therefore, the presence or absence of deep vein thrombosis could not be objectively established, and a venous thrombotic source was not documented. No dedicated diagnostic imaging for pulmonary embolism was performed.

During his stay in the intensive care unit, transthoracic echocardiography showed preserved left ventricular systolic function with a left ventricular ejection fraction of 58%, normal diastolic function, and no regional wall-motion abnormalities. The left atrium was normal in size, and no intracardiac thrombi or masses were identified by this method. The mitral valve showed no stenosis or regurgitation. Right ventricular systolic function was reduced (tricuspid annular plane systolic excursion (TAPSE) 14 mm and fractional area change 23%), and pulmonary artery systolic pressure was estimated at 44 mmHg. Agitated saline contrast demonstrated a significant right-to-left intracardiac shunt, with more than 30 microbubbles appearing in the left-sided cardiac chambers from the second cardiac cycle.

Transesophageal echocardiography was subsequently performed and confirmed a PFO with a hypermobile interatrial septum showing an excursion of 9 mm, without meeting criteria for an atrial septal aneurysm. The PFO tunnel measured approximately 5 mm in length and 4 mm in thickness. The septum secundum measured 5 mm and the septum primum 1 mm. Specific assessment of the left atrial appendage for thrombus was not documented in the available report. The echocardiographic findings are shown in Figure 3. 

**Figure 3:**
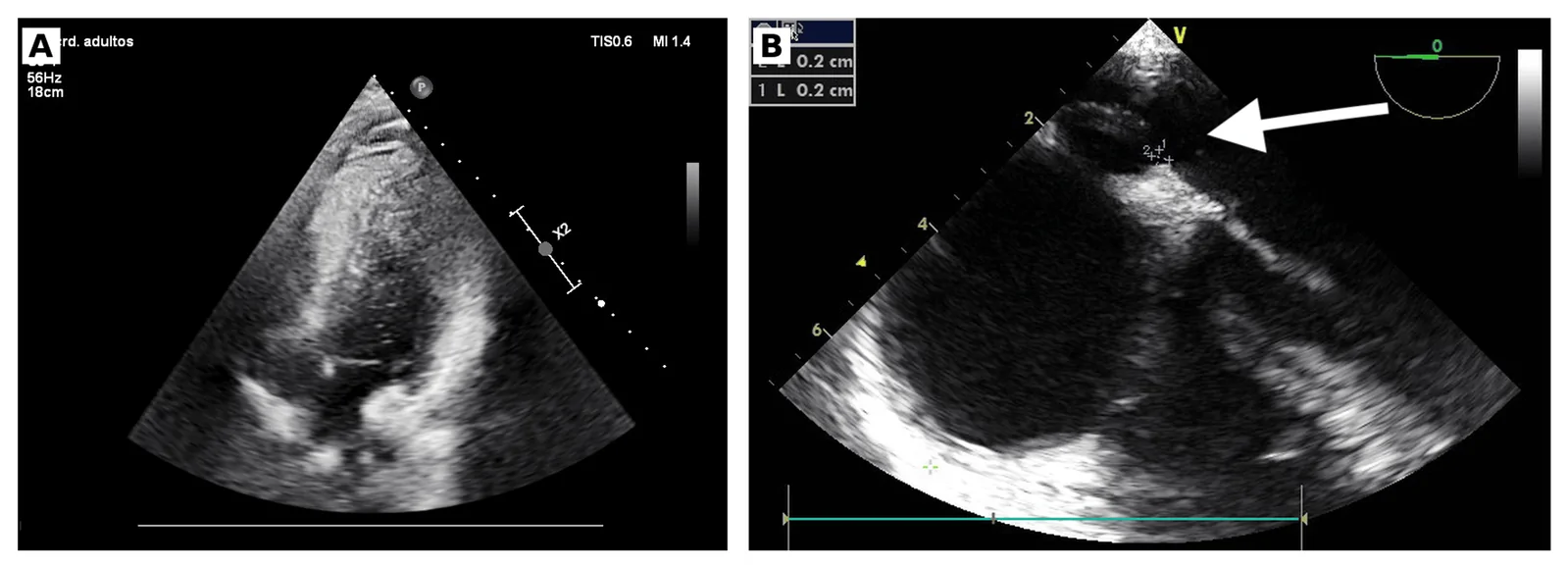
Echocardiographic evaluation of the patent foramen ovale (A) Transthoracic echocardiography with agitated saline contrast demonstrating passage of microbubbles into the left-sided cardiac chambers, consistent with a right-to-left intracardiac shunt. (B) Transesophageal echocardiography demonstrating the patent foramen ovale and the interatrial septum. The arrow indicates the site of anatomical measurement.

Retrospective assessment yielded a Risk of Paradoxical Embolism (RoPE) score of 9 [6]. PFO closure was not performed during the index hospitalization, and the patient continued monthly follow-up with cardiology for evaluation of definitive management. At hospital discharge, antithrombotic therapy consisted of apixaban 5 mg twice daily and aspirin 100 mg once daily, without a predefined discontinuation date at the time of discharge.

Follow-up brain CT showed no intracranial hemorrhage or significant cerebral edema (Figure 4). The patient had a favorable neurological course, and after normal findings on noninvasive neuromonitoring, mechanical ventilation was discontinued and rehabilitation was started. At hospital discharge, the NIHSS score was 8, attributable to the pre-existing bilateral lower-extremity paralysis associated with the traumatic spinal cord injury. No residual neurological deficits attributable to the ischemic stroke were identified, while the deficits related to the TSCI persisted. Follow-up with neurology, neurosurgery, and rehabilitation was continued.

**Figure 4:**
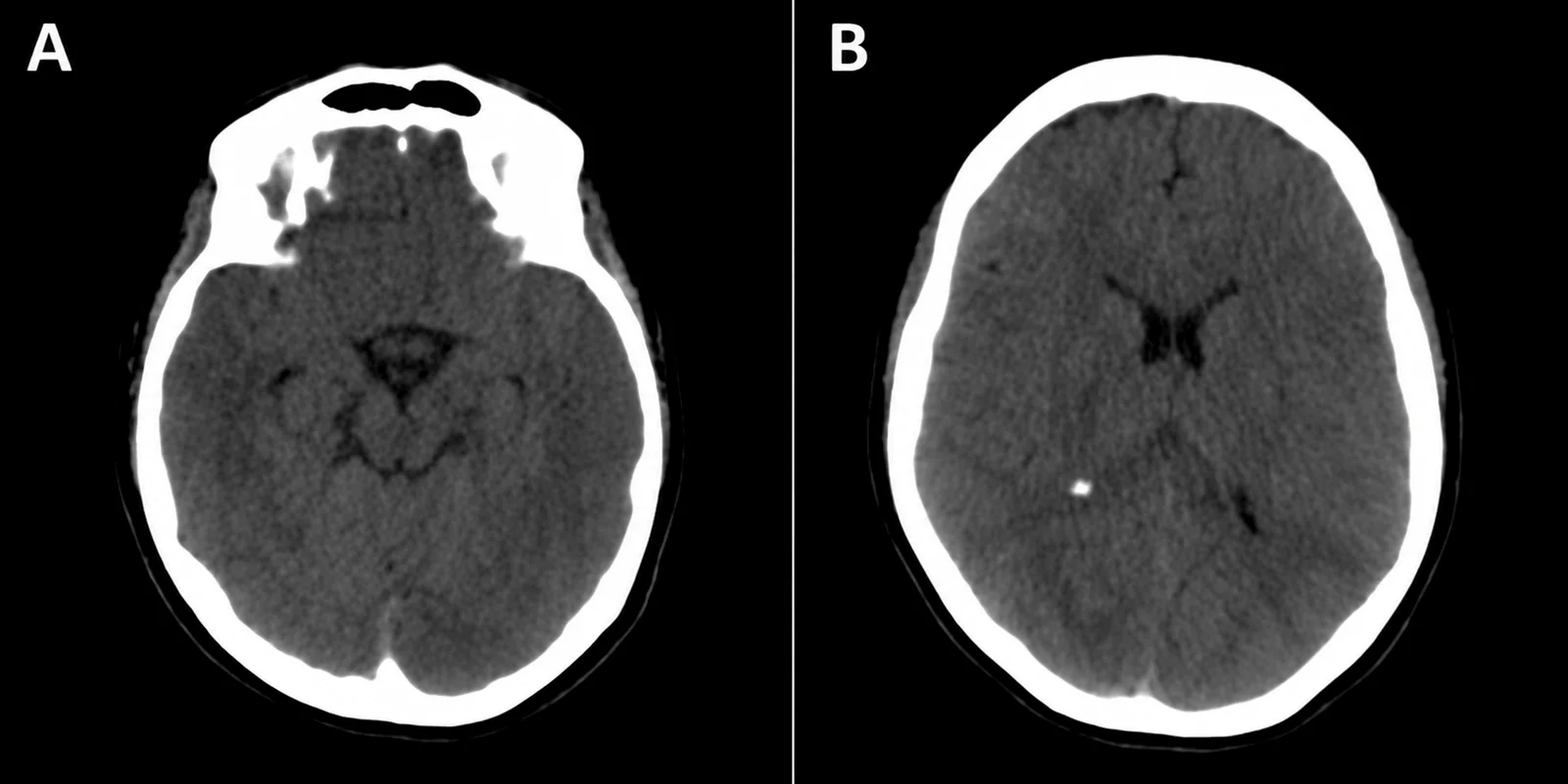
Follow-up brain CT (axial view) after mechanical thrombectomy. (A) Image at the level of the posterior fossa and (B) supratentorial image showing no evidence of intracranial hemorrhage or significant cerebral edema.

A summary of the clinical course is presented in Figure 5.

**Figure 5:**
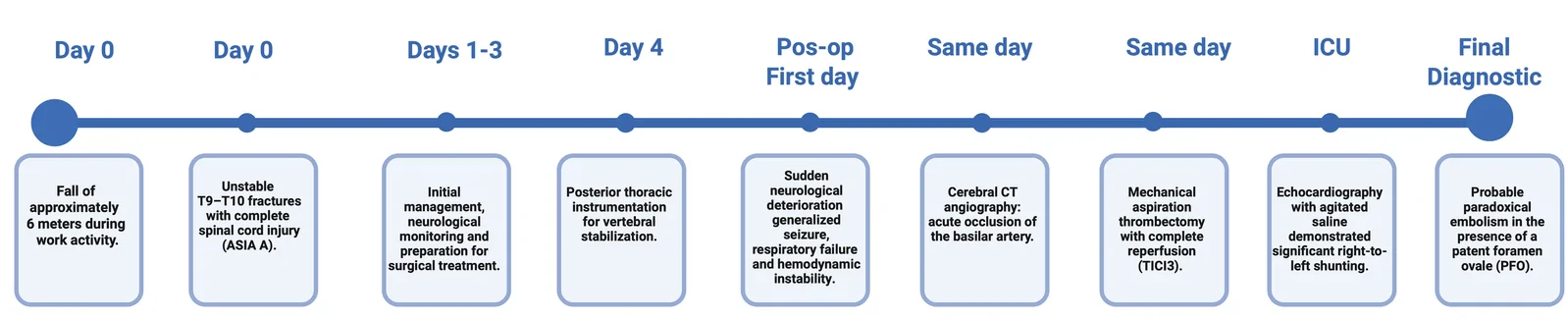
Clinical timeline of the case. On hospital day 0 the patient sustained a 6-meter fall resulting in T9–T10 fractures and complete traumatic spinal cord injury (ASIA A). Initial management and neurological monitoring continued during hospital days 1–3. Posterior thoracic instrumentation was performed on hospital day 4. On the first postoperative day, the patient developed abrupt neurological deterioration with generalized seizure activity, respiratory failure, and hemodynamic instability. Emergency computed tomography and angiography demonstrated acute distal basilar artery occlusion followed by mechanical thrombectomy with complete reperfusion (TICI 3) on the same day. Subsequent transthoracic echocardiography with agitated saline demonstrated significant right-to-left shunting, and transesophageal echocardiography confirmed a patent foramen ovale (PFO), supporting paradoxical embolism as a probable mechanism of the cerebrovascular event. ASIA: American Spinal Injury Association; TICI: thrombolysis in cerebral infarction scale; pos-op: postoperative Image created by authors using BioRender (https://BioRender.com/ibw9gse)

## Discussion

Acute basilar artery occlusion is one of the most severe forms of ischemic stroke. Although it accounts for only 1-4% of all ischemic strokes, it is associated with high mortality and disability when timely reperfusion is not achieved. In recent years, several clinical trials have changed the treatment of basilar artery occlusion by showing that mechanical thrombectomy can improve functional outcomes in selected patients with posterior circulation occlusion. It is now considered an important reperfusion strategy in appropriately selected patients [1-6].

Our patient presented a particularly complex scenario because the stroke occurred during hospitalization for a severe TSCI that had recently been treated with surgical stabilization. Intravenous thrombolysis was not considered a therapeutic option in this setting because of the recent major trauma and spinal surgery, and thus mechanical thrombectomy was performed. Direct aspiration achieved complete reperfusion (TICI 3), followed by a favorable neurological course during his stay in the intensive care unit. This is consistent with the available evidence supporting endovascular reperfusion in selected patients with basilar artery occlusion [3-6].

The etiology of the ischemic event is one of the most relevant aspects of this case. As part of the etiological workup, transthoracic echocardiography with agitated saline showed a right-to-left shunt with more than 30 microbubbles appearing in the left-sided cardiac chambers from the second cardiac cycle. Transesophageal echocardiography was then performed and showed a PFO with increased interatrial septal mobility of 9 mm, without meeting criteria for an atrial septal aneurysm. These findings provided an anatomical pathway that could allow embolic material to pass from the venous to the arterial circulation [8,9].

Paradoxical embolism is a recognized mechanism of ischemic stroke in patients with PFO. A thrombus originating in the venous system may cross a PFO during a transient or persistent right-to-left shunt and subsequently reach the cerebral arterial circulation. The presence of a significant shunt and certain anatomical features of the PFO may increase the likelihood that the PFO is related to the stroke rather than being an incidental finding in a young patient [8,9]. In our patient, the early passage of more than 30 microbubbles and the increased mobility of the interatrial septum supported the possibility of this mechanism.

Several factors that increase the risk of venous thrombus formation were present in our patient, including major trauma, complete spinal cord injury, prolonged immobilization, and recent surgery [10,11]. These factors can be interpreted within the framework of Virchow’s triad. Complete spinal cord injury and lower-extremity paralysis reduce the skeletal-muscle pump and promote venous stasis, while major trauma and recent surgery may contribute to endothelial injury and a transient inflammatory and hypercoagulable state. Importantly, the risk of VTE after acute spinal cord injury is highest during the early post-injury period, particularly within the first weeks after injury [10]. Therefore, the neurological event occurring on day 5 in our patient occurred during a period of substantial thromboembolic risk. However, because no venous thrombus was objectively documented, this case cannot be considered evidence of an early VTE event or claimed to represent the earliest VTE reported after spinal cord injury. 

In the presence of a PFO with significant right-to-left shunting, a venous thrombus could potentially bypass the pulmonary circulation and enter the systemic arterial circulation, providing a plausible pathway for cerebral paradoxical embolism [8,9]. Although the presence or absence of deep venous thrombosis could not be objectively established because dedicated venous imaging was not performed, the combination of this high-risk clinical setting and the PFO made paradoxical embolism a plausible mechanism. However, the absence of a documented venous thrombotic source prevents this mechanism from being established with certainty. The proposed mechanism of paradoxical embolism in this case is summarized in Figure 6.

**Figure 6:**
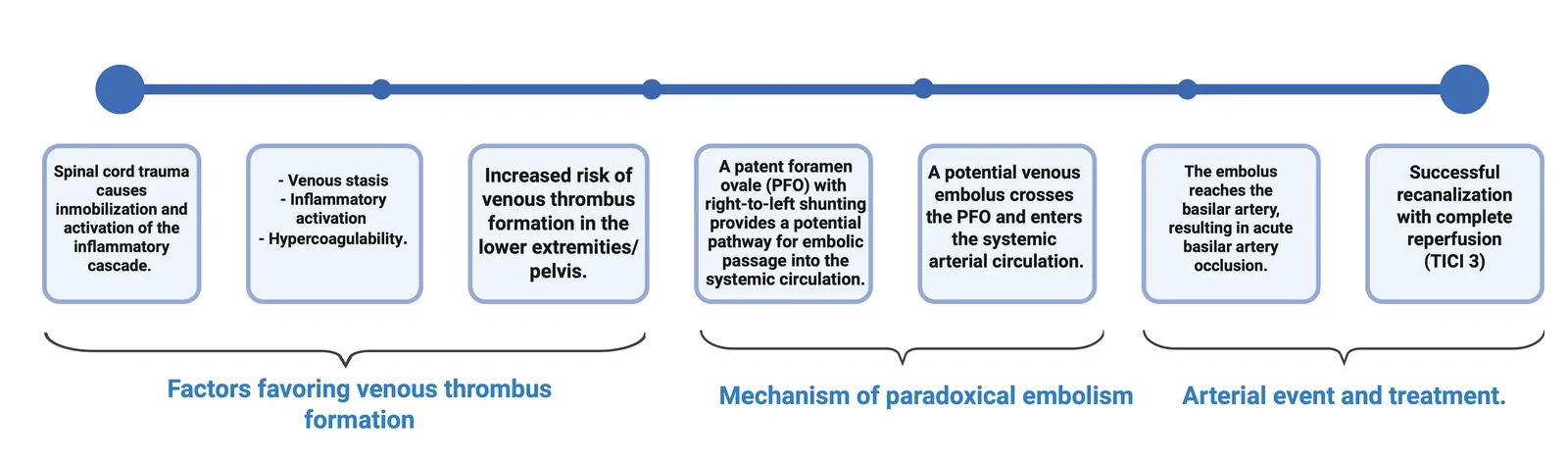
Proposed mechanism of paradoxical embolism. Traumatic spinal cord injury with immobilization increases the risk of venous thrombosis formation. In the presence of a patent foramen ovale (PFO) with right-to-left shunting, a venous embolus may enter the systemic arterial circulation and reach the posterior cerebral circulation, resulting in basilar artery occlusion. Image created by authors using BioRender (https://BioRender.com/n6kpp8o)

Similar presentations of posterior circulation ischemic stroke associated with PFO and suspected paradoxical embolism have been reported, although basilar artery involvement appears to be uncommon. Previous reports have described basilar or vertebrobasilar ischemic events in young patients in whom a PFO was identified during the etiological workup. As in the present case, these reports emphasize that identification of a PFO alone does not establish causality and that competing stroke mechanisms should be investigated. A distinctive feature of our case is the occurrence of basilar artery occlusion in the setting of complete TSCI, recent spinal surgery, and immobilization, providing a substantial clinical substrate for venous thrombus formation despite the absence of documented deep venous thrombosis [17].

Alternative etiologies of basilar artery occlusion were also considered. CTA showed no evidence of traumatic arterial injury. A cardioembolic mechanism related to atrial fibrillation was less supported because electrocardiography and continuous cardiac rhythm monitoring showed sinus rhythm without documented arrhythmias; however, prolonged Holter monitoring was not performed, and paroxysmal atrial fibrillation cannot be completely excluded. Transthoracic and transesophageal echocardiographic evaluation showed no intracardiac masses or thrombi, making a structural cardiac source such as myxoma less likely. Polycythemia was not present (hemoglobin 12.3 g/dL and hematocrit 34%). Routine coagulation studies were obtained; however, specific testing for inherited or acquired thrombophilia, including protein C, protein S, antithrombin III, factor V Leiden, antiphospholipid antibodies, lupus anticoagulant, and homocysteine, was not performed. No known history or clinical findings suggesting malignancy were identified during hospitalization, although systematic malignancy screening was not performed. Therefore, these alternative thrombotic sources cannot all be completely excluded. The PFO with significant right-to-left shunting [8,9], together with the marked venous thromboembolic risk associated with trauma, spinal cord injury, surgery, and immobilization [10-12], supported paradoxical embolism as the most plausible rather than a definitively established mechanism.

This case has several strengths, including in-hospital recognition of stroke onset, precise documentation of the acute treatment timeline, angiographic confirmation of basilar artery occlusion and complete reperfusion, and detailed echocardiographic characterization of the PFO and right-to-left shunt. Important limitations should also be acknowledged. Lower-extremity venous Doppler ultrasonography or CT venography was not performed; therefore, the presence or absence of deep vein thrombosis could not be objectively established. Prolonged ambulatory rhythm monitoring with Holter ECG was also not performed. Specific testing for inherited or acquired thrombophilia was not performed. Furthermore, the presence of a PFO does not by itself establish a causal relationship with the ischemic event. Despite these limitations, the combination of the cardiac findings and the markedly prothrombotic clinical setting supports paradoxical embolism as the most plausible mechanism in this case, while recognizing that it cannot be established as the definitive cause.

## Conclusions

This case describes acute basilar artery occlusion in a young patient hospitalized for severe TSCI who achieved complete reperfusion after mechanical thrombectomy. The presence of a PFO with significant right-to-left shunting, together with major trauma, recent surgery, and prolonged immobilization, raised the possibility of paradoxical embolism. However, because a venous thrombotic source was not objectively documented and the etiological evaluation did not completely exclude all alternative thromboembolic mechanisms, a causal relationship between the PFO and the basilar artery occlusion cannot be definitively established. This case highlights the importance of prompt recognition and endovascular treatment of acute neurological deterioration in hospitalized patients and of a systematic etiological evaluation in young patients with ischemic stroke.
